# Molecular basis of quinoa’s resilience to abiotic stresses: implications for climate-adaptive crop breeding

**DOI:** 10.3389/fpls.2026.1865530

**Published:** 2026-07-08

**Authors:** Shimei Tan, Yudan Xiang, Anfel Soltani, Mingguang Lei

**Affiliations:** College of Life and Environmental Sciences, Hangzhou Normal University, Hangzhou, China

**Keywords:** abiotic stress, molecular breeding, quinoa, stress signaling, transcription factors

## Abstract

Quinoa (*Chenopodium quinoa* Willd.) has emerged as a compelling model for understanding plant resilience to environmental adversity. As a facultative halophyte native to the Andean highlands, quinoa tolerates drought, salinity, temperature extremes, and nutrient-poor soils through a multi-layered molecular defense system that is both conserved with other plants and enriched with quinoa-specific innovations. This review synthesizes current knowledge of three interconnected regulatory tiers that underpin quinoa’s stress resilience. First, a suite of functional proteins provides the immediate cellular defense against stress-induced damage. Second, a diverse repertoire of transcription factor families orchestrates the transcriptional reprogramming required for stress adaptation, with several families showing quinoa-specific expansions and functionally validated members. Third, interconnected signaling networks integrate stress perception with adaptive responses through extensive crosstalk and feedback regulation. We further highlight how multi-omics approaches are revealing stress-specific regulatory hubs and genotype-dependent adaptive strategies. Finally, we identify critical knowledge gaps and propose research priorities that will be essential for translating mechanistic insights into climate-adaptive crop improvement.

## 1 Introduction

Originating from the Andean highlands of South America, quinoa (*Chenopodium quinoa* Willd.) has gained global recognition not only for its superior nutritional composition but also for its remarkable capacity to withstand adverse environmental conditions (Alandia et al., 2020; Hinojosa et al., 2018). This allotetraploid pseudocereal provides a nutritionally complete amino acid profile, with particularly elevated concentrations of lysine and methionine that are typically limiting in cereal crops, and is a rich source of minerals, vitamins, and bioactive compounds (Angeli et al., 2020; Pathan and Siddiqui, 2022). Its gluten-free nature and high protein content have driven rapidly expanding global demand, positioning quinoa as a strategically important crop for food security and human nutrition (Filho et al., 2017).

The urgency of developing stress-resilient crops has never been greater. Climate change projections forecast a significant increase in the frequency and severity of drought events, rising temperatures, and expanding soil salinization, all of which threaten global agricultural productivity (Hinojosa et al., 2018). Against this backdrop, quinoa occupies a unique position. It is simultaneously a nutritionally superior food crop and a genetic reservoir of stress-tolerance mechanisms that are not fully represented in major cereal crops. Historical records document that pre-Columbian Andean societies successfully cultivated quinoa at elevations approaching 4,000 m above sea level, where plants endured pronounced diurnal temperature fluctuations, intense UV radiation, and periodic drought (Jacobsen et al., 2015). This evolutionary history has endowed quinoa with a molecular toolkit that is of direct relevance to climate-adaptive crop breeding. Understanding the genetic and molecular bases of quinoa’s resilience therefore offers an opportunity not only to elucidate fundamental principles of plant stress adaptation, but also to identify candidate genes, regulatory networks, and breeding targets that can be deployed to improve stress tolerance in quinoa itself and, potentially, in related crops.

**Figure 1:**
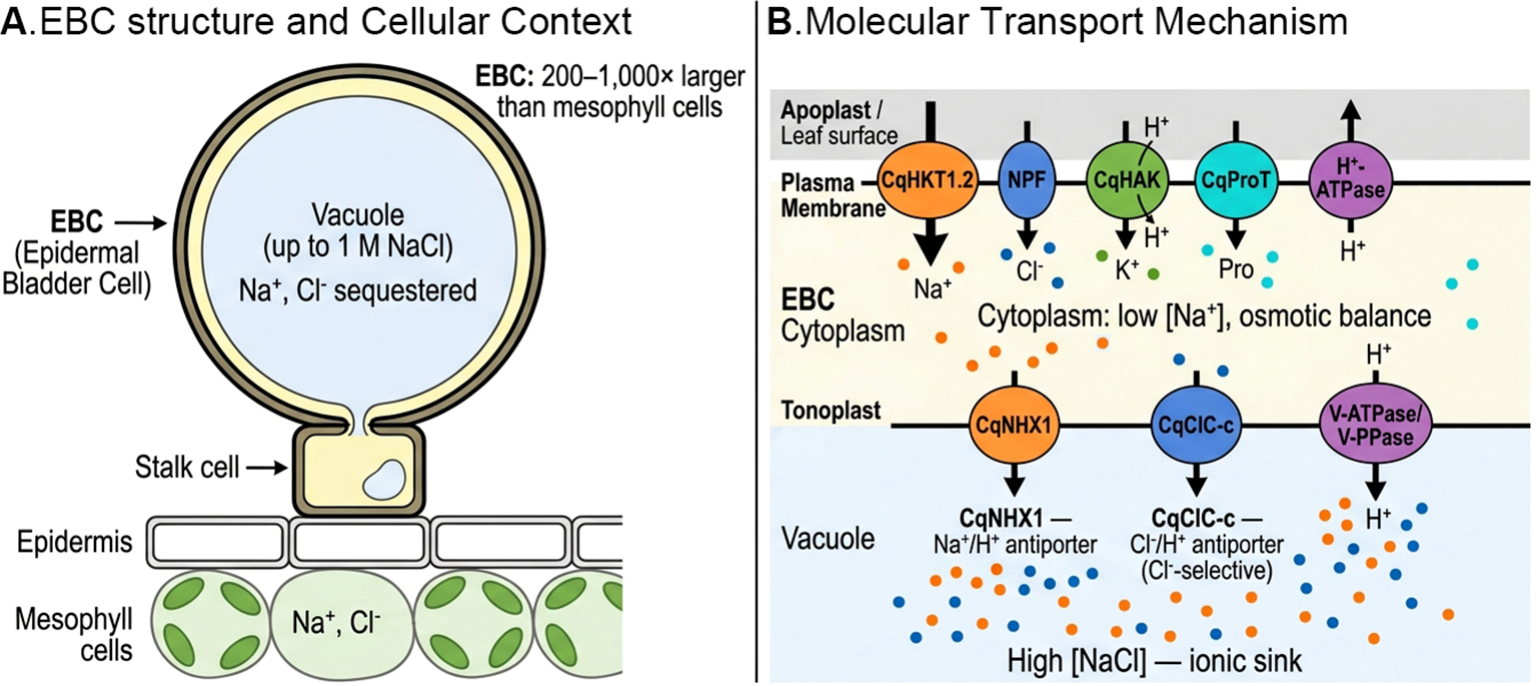
Ion compartmentalization in quinoa epidermal bladder cells (EBCs) and its functional significance for salinity tolerance. **(A)** Cross-sectional view of a quinoa leaf showing an EBC attached to the epidermis via a morphologically distinct stalk cell. The EBC is 200–1,000× larger than mesophyll cells and accumulates Na^+^ and Cl^-^ in its giant central vacuole, physically sequestering toxic ions away from photosynthetically active tissues. **(B)** Molecular transport model of the EBC. The constitutive expression of ion transporters in EBCs is a key functional distinction from glycophytes, where transporter expression is typically stress-inducible. At the plasma membrane, CqHKT1.2 functions as a voltage-gated Na^+^ inward channel; NPF transporters mediate Cl^-^ uptake; CqHAK imports K^+^; and CqProT facilitates compatible solute accumulation. At the tonoplast, CqNHX antiporters drive Na^+^ into the vacuole, energized by V-ATPase and V-PPase proton pumps.

Quinoa employs multiple, often interconnected physiological strategies to counteract environmental stresses. Under water deficit, it reduces transpirational water loss through stomatal regulation while simultaneously exploiting its well-developed root architecture to access subterranean water reserves (Ain et al., 2023; Nguyen et al., 2022). A notable anatomical adaptation is the presence of specialized epidermal bladder cells (EBCs) on leaf and stem surfaces (**Figure 1A**), which fulfill dual protective roles: sequestering toxic Na^+^ and Cl^-^ ions away from photosynthetically active mesophyll cells under salinity, and acting as putative water reservoirs that buffer against dehydration under drought (Kiani-Pouya et al., 2017; Otterbach et al., 2021). These EBCs are 200–1,000× larger than mesophyll cells and represent one of the most distinctive structural adaptations among halophytic crops, setting quinoa apart from most other salt-tolerant species including *Atriplex* spp (Böhm et al., 2018; Kiani-Pouya et al., 2017, 2020).

Quinoa also displays considerable phenotypic plasticity in response to environmental cues (Jacobsen et al., 2015; Murphy et al., 2016). Some cultivars can accelerate their developmental cycles under shortened photoperiods to circumvent peak drought periods, while others shed leaves to reduce transpirational surface area under severe water deficit (Ain et al., 2023). Despite this broad tolerance, quinoa’s capacity to withstand sustained high temperatures remains relatively modest compared with its salinity and drought tolerance, highlighting the importance of understanding the molecular basis of stress-specific responses and their interactions (Grenfell-Shaw and Tester, 2021).

This review comprehensively examines the genetic and molecular bases of quinoa’s responses to major abiotic stresses, including drought, salinity, heat, cold, and waterlogging. We analyze how quinoa maintains cellular function under stress through the coordinated activation of genes governing osmotic adjustment, antioxidant protection, and ion homeostasis. We also incorporate recent discoveries that have substantially broadened our understanding of quinoa stress adaptation, including newly characterized transcription factor families, signaling pathway components, and multi-omics datasets. Throughout, we highlight what is genuinely quinoa-specific versus what represents conserved plant stress biology, and we identify the most pressing knowledge gaps that future research must address.

## 2 Key functional proteins in quinoa abiotic stress response

**Figure 2:**
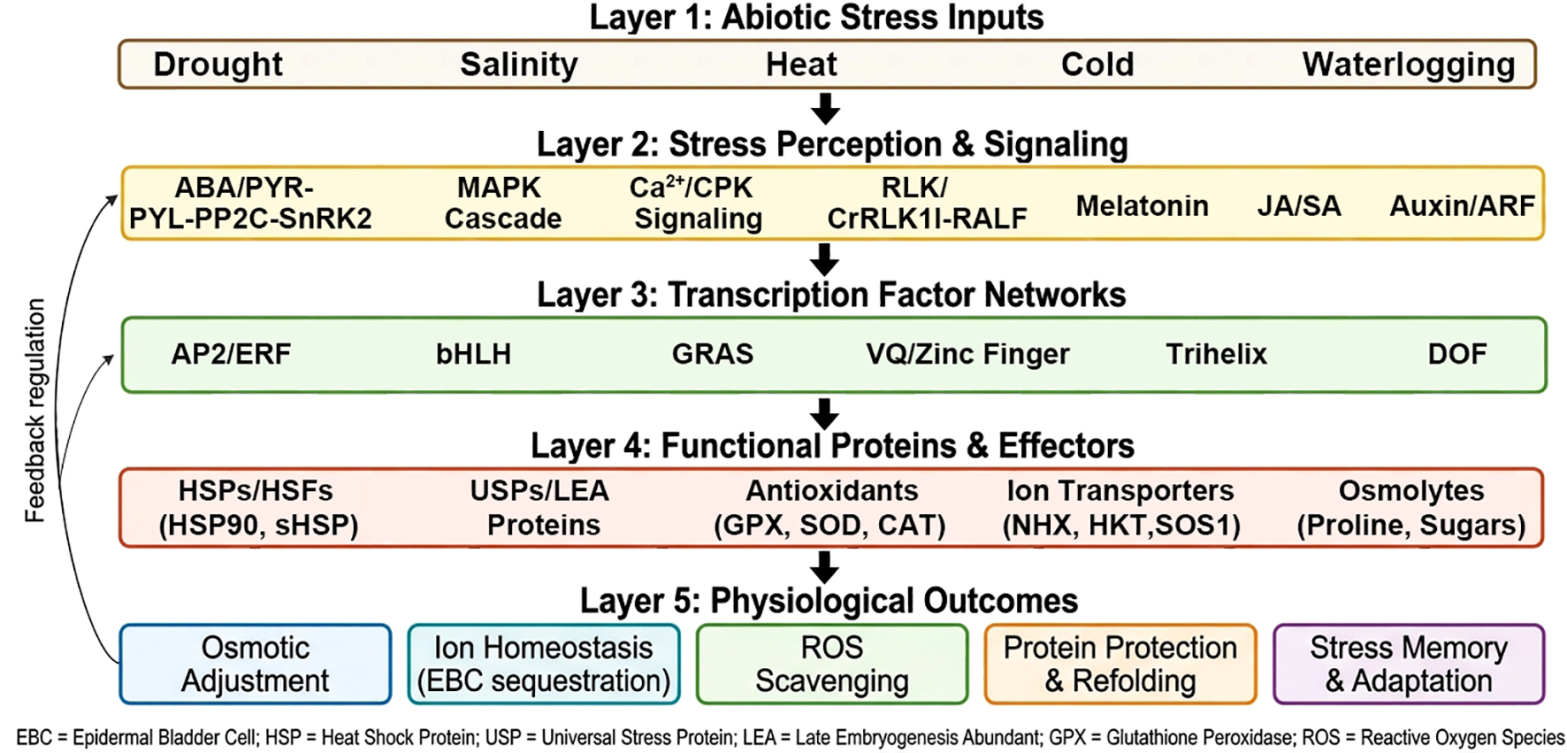
Conceptual model of molecular mechanisms underlying quinoa responses to multiple abiotic stresses. This figure presents an integrated framework synthesizing evidence from quinoa and related plant species. The diagram is organized as a five-layer hierarchical framework. Layer 1 (top): five major abiotic stresses (drought, salinity, heat, cold, and waterlogging) that quinoa encounters in its native and cultivated environments. Layer 2: stress perception and signaling pathways, including the hormone-mediated cascades (ABA/PYR-PYL-PP2C-SnRK2, auxin/ARF, JA/SA, melatonin) and non-hormone cascades (MAPK, Ca²^+^/CPK, RLK/CrRLK1L-RALF). Layer 3: transcription factor networks (AP2/ERF, bHLH, GRAS, VQ, Trihelix, DOF, COL, ARF, ZAT) that translate signaling inputs into transcriptional reprogramming. Layer 4: functional protein outputs, including HSPs/USPs, ion transporters/aquaporins, and antioxidant enzymes/LEA proteins. Layer 5 (bottom): physiological outcomes that collectively determine stress resilience.

Environmental stress triggers the activation of a complex molecular network in quinoa, comprising proteins that mediate osmoprotection, antioxidant defense, and maintenance of ionic equilibrium. These functional proteins represent the first line of cellular defense against stress-induced damage and operate in a highly coordinated manner (**Figure 2**). Critically, while many of these protein families are broadly conserved across the plant kingdom, quinoa exhibits notable specializations — including EBC-specific transporter expression, an unusually large glutathione peroxidase family, and co-induction of heat shock and universal stress proteins under overlapping stresses — that distinguish its stress response from that of glycophytic crops.

### 2.1 Heat shock proteins and stress protection proteins

Heat shock proteins (HSPs) serve as essential molecular chaperones in quinoa’s adaptive response to heat and other environmental stresses. In plants generally, HSPs recognize and bind to hydrophobic domains exposed during stress-induced protein denaturation, thereby preventing misfolding and irreversible aggregation and preserving proteome integrity (Jacob et al., 2017; Al-Whaibi, 2011). In quinoa specifically, the predominant HSP families comprise HSP70, HSP90, and small heat shock proteins (sHSPs), each fulfilling distinct chaperone functions.

A genome-wide survey identified 11 HSP90 family members in quinoa, each exhibiting distinct expression profiles under heat stress conditions (Chen et al., 2025). Notably, quinoa HSP90 genes show closer evolutionary relationships to dicotyledonous species such as *Arabidopsis thaliana* and tomato (*Solanum lycopersicum*) than to monocots, consistent with quinoa’s taxonomic placement within the Caryophyllales. Transgenic validation in *Arabidopsis* confirmed that CqHSP90.1c and CqHSP90.6a function as positive regulators of thermotolerance (Chen et al., 2025).

The HSP70 subfamily has also been comprehensively characterized: 16 Cqhsp70 members were identified, forming eight paralogous pairs that reflect quinoa’s allopolyploid origin (Liu et al., 2018). These members exhibit diverse subcellular localizations, suggesting specialized chaperone functions across cellular compartments. Under drought stress, expression profiling revealed heterogeneous response patterns. *AUR62004581* (a plastid-localized member) showed the most dramatic induction, while *AUR62024018* maintained sustained high expression throughout the stress period. Approximately half of the analyzed members displayed a characteristic ‘drop-climb-drop’ kinetic pattern consistent with their *Arabidopsis* orthologs (Liu et al., 2018).

In addition to HSPs, the Universal Stress Protein (USP) family provides a complementary layer of protection. USPs function cooperatively with HSPs to preserve cellular homeostasis when plants encounter multiple concurrent stresses — a scenario increasingly common under field conditions. Genome-wide characterization of the *CqUSP* gene family revealed their involvement in ROS detoxification and cellular defense under drought and heat stress conditions (Imran et al., 2025). The co-induction of *HSPs* and *USPs* under overlapping stresses suggests a coordinated chaperone network that may confer broader multi-stress resilience than either family alone, though direct functional interaction between CqHSPs and CqUSPs in quinoa remains to be experimentally demonstrated.

The immediate transcriptional response to heat stress is governed by the heat shock factor (HSF)–HSP regulatory cascade. HSFs, particularly HSFA1s, form trimeric complexes that bind heat shock elements (HSEs) in target gene promoters, initiating active transcription of downstream HSP genes upon stress perception (Ohama et al., 2017; Andrási et al., 2021). Temporal expression analyses in quinoa demonstrated a sequential activation pattern: *CqHsf3* and *CqHsf9* transcripts were elevated at 6 h post-heat treatment, while *CqHsf4* and *CqHsf10* showed higher expression at 12 h, suggesting a transcriptional relay that sustains the heat stress response over time (Tashi et al., 2018).

### 2.2 Ion transporters, aquaporins, and the EBC system

Quinoa’s ability to flourish in saline habitats depends on complex ion regulatory systems that prevent toxic ion accumulation in the cytoplasm while maintaining osmotic balance (Grenfell-Shaw and Tester, 2021). The most distinctive feature of quinoa’s salt tolerance strategy is its EBC system. EBCs are connected to the epidermis via a morphologically distinct stalk cell, and constitutively express a suite of ion transporters regardless of salinity status (Zou et al., 2017; **Figure 1B**). This constitutive expression pattern contrasts sharply with the stress-inducible transporter expression seen in most glycophytes and even in other halophytes such as *Atriplex* spp., which rely primarily on vacuolar sequestration within mesophyll cells rather than dedicated epidermal storage structures (Kiani-Pouya et al., 2017). The EBC system thus represents a genuinely quinoa-enriched adaptation, though analogous structures exist in some other Chenopodiaceae members.

At the molecular level, EBCs express *CqHKT1.2* as a voltage-gated Na^+^ inward channel, NPF transporters for Cl^-^ uptake, *CqHAK* for K^+^ import, and *CqProT* for compatible solute accumulation (Böhm et al., 2018). Vacuolar sequestration is driven by CqNHX antiporters and energized by V-ATPase and V-PPase proton pumps. Systematic genome-wide analysis characterized 10 NHX family members (CqNHX1–10) distributed across vacuolar, plasma membrane, and endosomal compartments, with vacuolar NHXs showing the highest induction in leaf tissues under salinity stress (Santhoshi et al., 2025). Functional characterization further demonstrated that CqHKT1 and CqSOS1 mediate genotype-specific Na^+^ exclusion under high salinity, with expression levels correlating with salinity tolerance across quinoa accessions (Kobayashi et al., 2025). This genotype-dependent variation in transporter expression provides a molecular basis for the phenotypic diversity in salt tolerance observed across quinoa germplasm, and highlights CqHKT1 and CqSOS1 as priority targets for marker-assisted breeding.

### 2.3 Antioxidant defense system

Environmental stresses trigger excessive accumulation of reactive oxygen species (ROS), which inflict oxidative damage on lipids, proteins, and nucleic acids, thereby compromising cellular integrity (Gill and Tuteja, 2010; Mittler et al., 2022). To counteract this oxidative threat, quinoa has evolved an antioxidant defense system comprising both enzymatic components — superoxide dismutase (SOD), catalase (CAT), peroxidase (POD), and glutathione peroxidase (GPX) — and non-enzymatic antioxidants including ascorbate, glutathione, flavonoids, and carotenoids. A particularly notable feature of quinoa’s antioxidant system is the expansion of the GPX family: genome-wide screening identified 15 *CqGPX* genes, compared with 8 in *Arabidopsis thaliana*, suggesting that gene family expansion may have contributed to quinoa’s enhanced oxidative stress tolerance (Yang et al., 2025). Expression profiling revealed that multiple *CqGPX* members are transcriptionally induced by drought (PEG), salinity (NaCl), and oxidative stress (H_2_O_2_) treatments, with distinct patterns consistent with roles in both cytosolic and chloroplastic ROS detoxification (Yang et al., 2025). Notably, heterologous expression of *CqGPX4* and *CqGPX15* in *E. coli* conferred stress tolerance, providing initial functional evidence for their protective roles (Yang et al., 2025).

The antioxidant system operates in concert with complementary stress-protective mechanisms. Late embryogenesis abundant (LEA) proteins potentiate antioxidant defenses by stabilizing cellular structures under dehydration, while USPs contribute to ROS scavenging as part of their broader stress-protective functions (Imran et al., 2025). Drought-tolerant quinoa genotypes consistently maintain higher activities of SOD, CAT, and POD, with lower accumulation of ROS, compared with sensitive genotypes, linking antioxidant capacity directly to field-relevant stress tolerance (Zhu et al., 2024). Together, this coordinated network ensures efficient neutralization of stress-generated ROS and preservation of cellular redox balance across diverse stress conditions.

## 3 Transcriptional regulation of stress responses

Transcription factors (TFs) serve as key regulators of quinoa’s adaptive responses to environmental stresses, controlling the expression of downstream stress-inducible genes through sequence-specific DNA binding and protein–protein interactions. Genome-wide studies have characterized numerous TF families in quinoa, revealing both conserved regulatory mechanisms shared with other plant species and quinoa-specific adaptations (**Figure 2**). Rather than operating independently, these TF families form an interconnected regulatory network: AP2/ERF and bHLH factors frequently co-regulate stress-responsive promoters, WRKY factors interact with VQ co-regulators to fine-tune target gene specificity, and ARF factors integrate hormone signals with transcriptional outputs. The following subsections describe the major TF families implicated in quinoa stress responses, and **Table 1** provides a consolidated overview of their key features.

**Table 1:** Summary of major transcription factor families in quinoa abiotic stress responses.

TF family	Members in Quinoa	Key stress	Validated gene	Downstream targets/ Regulated pathways	Known/Putative PTMs	Reference
AP2/ERF	AP2: 23; DREB: 49; ERF: 71; RAV: 3; Soloist: 4	Drought, salinity, cold	CqERF24, CqDREB02, CqDREB12, CqERF37, CqERF50, CqERF54, CqRAV02	Proline accumulation; stress-responsive gene expression; ABA-responsive genes; osmotic adjustment	Phosphorylation by MAPK and SnRK2 (putative, by analogy with Arabidopsis)	Zhu et al., 2023; Bakhtari et al., 2024
bHLH	218 (20 subgroups)	Cold, heavy metal (Cd), drought, salinity	CqbHLH162	CqNRAMP1 (Cd transporter); ICE1-CBF-COR cold acclimation pathway (putative); flavonoid biosynthesis	Not yet characterized in quinoa	Xue et al., 2023; Sun et al., 2025b
GRAS	54	Drought, salinity (putative)	None validated in quinoa	Gibberellin signaling; root morphogenesis; symbiotic associations (inferred from orthologs)	Not yet characterized in quinoa	Zhu et al., 2021; Hou et al., 2025
VQ	23	Drought	CqVQ13	WRKY-mediated stress-responsive gene regulation; drought-responsive gene networks	Not yet characterized in quinoa	Zhang et al., 2025
ZAT	8	Multiple abiotic stresses	None validated in quinoa	Transcriptional repression of stress-response genes; fine-tuning of stress activation to prevent growth penalties	Not yet characterized in quinoa	Alvarez-Vasquez et al., 2025
Trihelix	47	Salinity	Cqtrihelix23	GT-box-containing stress-responsive promoters; salt tolerance pathways (downstream targets uncharacterized)	Not yet characterized in quinoa	Li et al., 2022; Sun et al., 2024
DOF	Not fully enumerated	Cold	CqDOF27	Flavonoid biosynthesis pathway (suppression); cold tolerance regulation	Not yet characterized in quinoa	Sun et al., 2025a
COL	Not fully enumerated	Multiple stresses (putative)	None validated in quinoa	Photoperiodic response; flowering time; stress-responsive gene expression (putative)	Not yet characterized in quinoa	Tran et al., 2025
ARF	26	Drought, salinity	CqARF05	AuxRE-containing auxin-responsive genes; root architecture; stress-responsive gene expression	Not yet characterized in quinoa	Wang et al., 2025a

### 3.1 AP2/ERF transcription factor family

The AP2/ERF superfamily consists of plant-specific transcription factors with a highly conserved AP2/ERF DNA-binding domain that recognizes GCC-box and DRE/CRT cis-elements in stress-responsive gene promoters. Genome-wide analyses have identified 148–150 *CqAP2/ERF* genes in the quinoa genome (Zhu et al., 2023; Bakhtari et al., 2024), classifiable into five subfamilies: AP2 (23 members), DREB (49), ERF (71), RAV (3), and Soloist (4). Segmental duplication was identified as the primary evolutionary driver, accounting for 64 genes (42.6%) and operating predominantly under purifying selection, indicating functional constraint on AP2/ERF gene copies following polyploidization (Bakhtari et al., 2024). Under salt stress, transcriptomic profiling identified 59 differentially expressed *CqAP2/ERF* genes, with *CqDREB36*, *CqDREB02*, and *CqERF29* most strongly upregulated in roots, and *CqERF37*, *CqDREB02*, and *CqERF30* most strongly upregulated in shoots (Bakhtari et al., 2024). RT-qPCR validation confirmed the salt-responsive expression of six members: *CqDREB02*, *CqDREB12*, *CqERF37*, *CqERF50*, *CqERF54*, and *CqRAV02* (Bakhtari et al., 2024). Functional characterization has revealed distinct roles for individual members: overexpression of *CqERF24* confers enhanced drought resistance through elevated proline accumulation and upregulation of stress-responsive genes, providing direct evidence that AP2/ERF factors translate stress signals into osmotic adjustment responses (Zhu et al., 2023). AP2/ERF members are also transcriptionally regulated by MAPK-mediated phosphorylation in other species, suggesting that post-translational modification may modulate their activity in quinoa as well, though this remains to be directly demonstrated.

### 3.2 bHLH transcription factor family

The basic helix-loop-helix (bHLH) family is one of the largest TF families in quinoa. Genome-wide characterization identified 218 *CqbHLH* genes, classified into 20 subgroups based on phylogenetic analysis and domain architecture (Xue et al., 2023). bHLH transcription factors regulate diverse aspects of plant development and stress adaptation, including iron homeostasis, flavonoid biosynthesis, stomatal differentiation, and cold acclimation through the ICE1-CBF-COR cascade. Expression profiling under multiple stress conditions revealed that numerous *CqbHLH* genes are differentially regulated, with cold-responsive members potentially participating in the ICE1-CBF pathway that is well-characterized in *Arabidopsis* (Xue et al., 2023). However, direct functional validation of individual CqbHLH members in quinoa stress responses has been limited. A notable exception is the recent characterization of *CqbHLH162* (ortholog of *AtbHLH38/101*), which forms a transcriptional complex with the MYB transcription factor *CqMYB26* to synergistically activate expression of the metal transporter gene *CqNRAMP1*, thereby promoting cadmium (Cd) uptake and sensitivity in quinoa (Sun et al., 2025b). Yeast one-hybrid and dual-luciferase assays confirmed direct binding of both *CqMYB26* and *CqbHLH162* to the *CqNRAMP1* promoter, while yeast two-hybrid and bimolecular fluorescence complementation assays demonstrated their protein-protein interaction. This MYB-bHLH dimer mechanism is well-established in anthocyanin biosynthesis regulation in model plants and extends to heavy metal stress responses in quinoa, providing the first direct functional evidence for a specific CqbHLH member in quinoa abiotic stress adaptation (Sun et al., 2025b). Functional characterization of additional CqbHLH members, particularly those implicated in drought and salinity responses, remains a priority for future research.

### 3.3 GRAS transcription factor family

The GRAS family comprises plant-specific transcription factors involved in diverse developmental and stress-responsive processes, named after the founding members GAI, RGA, and SCR. Fifty-four GRAS genes have been catalogued in the quinoa genome, including two recently characterized members with putative roles in stress responses (Zhu et al., 2021; Hou et al., 2025). GRAS proteins participate in gibberellin signaling, root morphogenesis, symbiotic associations, and light signaling pathways. Their involvement in quinoa stress responses is inferred primarily from expression data and phylogenetic relationships with functionally characterized orthologs in other species; direct functional validation in quinoa is still lacking for most family members.

### 3.4 VQ and zinc finger transcription factors

VQ (Valine-Glutamine) motif-containing proteins function as transcriptional co-regulators that physically interact with WRKY transcription factors to modulate their DNA-binding activity and target gene specificity. Twenty-three VQ family members have been identified in quinoa, with *CqVQ13* displaying pronounced upregulation under water deficit conditions, suggesting a role in drought-responsive gene regulation through WRKY-mediated pathways (Zhang et al., 2025). The zinc finger C2H2 (ZAT) family provides an additional layer of transcriptional control, with eight ZAT members identified in quinoa showing stress-responsive expression patterns (Alvarez-Vasquez et al., 2025). ZAT proteins typically function as transcriptional repressors that fine-tune stress responses by dampening excessive activation, thereby preventing growth penalties associated with constitutive stress gene expression.

### 3.5 Trihelix transcription factor family

The trihelix family includes transcription factors characterized by a conserved trihelix (helix-loop-helix-loop-helix) DNA-binding domain that recognizes GT-box elements in gene promoters. Forty-seven *Cqtrihelix* genes have been identified in the quinoa genome, with expression patterns suggesting roles in both development and stress responses (Li et al., 2022). Functional validation demonstrated that overexpression of *Cqtrihelix23* enhances salt tolerance in transgenic plants, providing direct evidence that at least one trihelix member contributes to quinoa’s salinity response (Sun et al., 2024). The downstream targets of Cqtrihelix23 and the mechanisms by which it confers salt tolerance — whether through regulation of ion transporter genes, osmolyte biosynthesis, or other pathways — remain to be elucidated.

### 3.6 DOF, COL, and ARF transcription factor families

The DOF (DNA Binding with One Finger) family includes CqDOF27, recently characterized as a cold-inducible transcription factor that paradoxically attenuates chilling tolerance through suppression of flavonoid accumulation (Sun et al., 2025a). This finding identifies flavonoid biosynthesis as a critical pathway for cold tolerance and suggests that targeted knock-down of *CqDOF27* could enhance quinoa’s cold resistance. Thus, *CqDOF27* is a potentially actionable target for gene editing. The CONSTANS-like (COL) family governs photoperiodic responses and flowering time. Genome-wide analysis of *CqCOL* genes revealed stress-responsive expression patterns that suggest roles beyond photoperiodism (Tran et al., 2025). The Auxin Response Factor (ARF) family mediates auxin-dependent transcriptional regulation through binding to auxin-responsive elements (AuxREs) in target gene promoters. Twenty-six ARF genes (*CqARF01*–*CqARF26*) have been catalogued in quinoa. Functional analyses demonstrated that *CqARF05* overexpression markedly enhances both drought and salt tolerance, likely by modulating root architecture and stress-responsive gene expression through auxin signaling (Wang et al., 2025a).

Despite the breadth of TF family characterizations in quinoa, important limitations must be acknowledged. The majority of studies to date have relied on genome-wide identification and expression profiling, with functional validation limited to a small number of individual genes (*CqERF24*, *Cqtrihelix23*, *CqARF05*, *CqDOF27*). The downstream target genes of most quinoa TFs remain uncharacterized, and the extent to which post-translational modifications — including phosphorylation by stress-activated kinases (SnRK2, MAPK), ubiquitin-mediated proteasomal degradation, and sumoylation — regulate TF activity in quinoa is largely unexplored. Furthermore, the combinatorial interactions between TF families — for example, AP2/ERF–bHLH co-regulation or WRKY–VQ complexes — represent key molecular regulatory nodes that have not yet been systematically investigated in quinoa. Addressing these gaps will require chromatin immunoprecipitation sequencing (ChIP-seq), protein interaction studies, and loss-of-function genetic analyses.

## 4 Stress signaling pathways

Quinoa perceives and transduces environmental stress signals through interconnected signaling cascades that coordinate physiological and molecular responses at multiple levels. These pathways form an integrated network characterized by extensive crosstalk, feedback regulation, and signal amplification (**Figure 2**). A key integrative node is the convergence of ABA and MAPK signaling on the SnRK2 kinase module: ABA-activated SnRK2s phosphorylate AREB/ABF transcription factors, while MAPK cascades independently activate overlapping sets of stress-responsive TFs, creating redundancy and robustness in the stress response. Calcium/CPK signaling serves as a shared second-messenger system that amplifies both hormone-dependent and hormone-independent stress signals, and melatonin functions as a molecular bridge between ROS homeostasis and hormone signaling networks. It is important to note that **Figure 2** represents a conceptual model integrating evidence from quinoa and related plant species.

### 4.1 Hormone-mediated signaling pathways

#### 4.1.1 ABA signaling pathway

Abscisic acid (ABA) acts as a master regulator of quinoa’s response to drought and osmotic stress. Stress-induced ABA accumulation is perceived by PYR/PYL receptor proteins, which inhibit PP2C phosphatases and thereby relieve SnRK2 kinases to phosphorylate downstream targets, including AREB/ABF transcription factors and guard cell ion channels that mediate stomatal closure (Chen et al., 2020). This canonical PYR/PYL-PP2C-SnRK2 module has been characterized in quinoa, where it coordinates stomatal regulation and stress-responsive gene expression under drought conditions (Pizzio, 2023; Morales et al., 2017). A recent study reported evidence suggesting that ABA additionally coordinates molecular networks integrating antioxidant defense, carbohydrate metabolism, and circadian clock components to confer tolerance to combined low-temperature and drought stresses (Lu et al., 2025), though the mechanistic basis of this multi-pathway integration requires further experimental validation. Importantly, transcriptomic analyses have also identified ABA-independent drought response pathways in quinoa (Morales et al., 2017), indicating that the stress response is not solely ABA-dependent and that multiple parallel signaling routes contribute to drought tolerance. Beyond the core SnRK2-AREB/ABF module, AP2/ERF transcription factors serve as important downstream effectors that translate ABA signals into transcriptional outputs. Specifically, CqDREB12 — the quinoa ortholog of DREB2C — is predicted to interact with ABF2, a key ABA-activated transcription factor, to co-activate ABA-responsive genes via DRE/CRT elements in their promoters (Bakhtari et al., 2024). CqRAV02 (ortholog of RAV1) provides a further mechanistic link: RAV1 is known to interact directly with ABI3 and ABI5, two central regulators of ABA-dependent gene expression during stress and seed maturation, and *CqRAV02* shows salt-responsive expression consistent with participation in this regulatory axis (Bakhtari et al., 2024). These connections between the ABA signaling cascade and AP2/ERF family members are consistent with the finding that 67.3% of *CqAP2/ERF* gene promoters contain ABRE elements (see Section 3.1), and collectively suggest that AP2/ERF TFs function as a transcriptional relay amplifying ABA signals under drought and salinity stress.

#### 4.1.2 Auxin signaling and the ARF family

Auxin signaling plays a significant role in both plant growth and stress responses by modulating root architecture, cell expansion, and stress-responsive gene expression. The ARF family mediates auxin-dependent transcriptional regulation through binding to AuxREs in target gene promoters. Twenty-six ARF genes (*CqARF01*–*CqARF26*) have been catalogued in quinoa, with functional analyses demonstrating that *CqARF05* overexpression markedly enhances both drought and salt tolerance, likely by modulating root architecture and stress-responsive gene expression (Wang et al., 2025a). The integration of auxin signaling with ABA pathways — through shared downstream targets and antagonistic interactions at the level of root growth regulation — suggests that the ARF family may serve as a node connecting growth-defense trade-offs in quinoa under stress.

#### 4.1.3 Jasmonic acid and salicylic acid signaling

Jasmonic acid (JA) and salicylic acid (SA) regulate defense and stress responses in quinoa through partially antagonistic yet functionally complementary signaling pathways. Transcriptomic profiling revealed distinct and largely non-overlapping transcriptional responses to exogenous methyl jasmonate (MeJA) and SA treatments, indicating that these hormones activate different downstream gene networks in quinoa (Rollano-Peñaloza et al., 2025). JA signaling promotes lignin biosynthesis, thereby reinforcing cell walls against mechanical stress and pathogen invasion, while SA activates pathogenesis-related (PR) gene expression and systemic acquired resistance. Under abiotic stress conditions, JA and SA signaling may interact with ABA pathways to fine-tune the balance between stress tolerance and growth, though the specific molecular mechanisms of this crosstalk in quinoa remain to be fully characterized.

#### 4.1.4 Melatonin biosynthesis and signaling

Melatonin functions as both a potent antioxidant molecule and a signaling compound in plant stress responses, scavenging ROS directly while also activating downstream defense gene expression. Genome-wide analysis identified 10 melatonin biosynthesis genes in quinoa: 3 TDCs (tryptophan decarboxylases), 2 T5Hs (tryptamine 5-hydroxylases), 3 SNATs (serotonin N-acetyltransferases), and 2 ASMTs (acetylserotonin O-methyltransferases), with expression of several members induced by salt and drought stress (Yolcu et al., 2025). Melatonin priming has been shown to enhance antioxidant enzyme activities and secondary metabolite production under drought stress in quinoa (Samadi et al., 2024). Mechanistically, melatonin acts as a molecular bridge between ROS homeostasis and hormone signaling: it suppresses ROS accumulation while simultaneously modulating ABA and JA signaling pathways, positioning it as an integrative regulator of multi-stress responses.

### 4.2 Non-hormone signaling cascades

#### 4.2.1 MAPK signaling cascade

Mitogen-activated protein kinase (MAPK) cascades relay stress signals from membrane receptors to downstream transcription factors and effector molecules through sequential phosphorylation of MAPKKK, MAPKK, and MAPK components. Comparative transcriptomic analyses identified MAPK signaling as a key pathway in quinoa’s response to cold stress during seed germination, with MAPK pathway genes significantly enriched among differentially expressed genes in cold-tolerant but not cold-sensitive cultivars (Fu et al., 2025). MAPK cascades also intersect with ABA signaling: in other plant species, MAPK-mediated phosphorylation of SnRK2 kinases and AP2/ERF transcription factors provides an ABA-independent route to stress gene activation, and a similar mechanism may operate in quinoa, though direct evidence is currently lacking.

#### 4.2.2 Calcium signaling and the CPK family

Calcium ions function as ubiquitous second messengers in plant stress responses, with stress-induced cytosolic Ca²^+^ transients decoded by calcium sensor proteins including calmodulins (CaMs), calmodulin-like proteins (CMLs), and calcium-dependent protein kinases (CPKs). CPKs directly phosphorylate downstream substrates to initiate stress-adaptive responses, and their activity is regulated by both Ca²^+^ binding and autophosphorylation. Computational characterization of the quinoa CPK family revealed salt-responsive expression profiles for multiple members (Lima-Huanca et al., 2025). Ca²^+^/CPK signaling intersects with both ABA and MAPK pathways: CPKs can phosphorylate AREB/ABF transcription factors downstream of ABA, and Ca²^+^ signals can activate MAPK cascades, creating a convergent signaling network that amplifies stress responses across multiple pathways.

#### 4.2.3 Receptor-like kinase signaling

Receptor-like kinases (RLKs) detect extracellular signals at the cell surface and trigger intracellular signaling cascades through their cytoplasmic kinase domains. The CrRLK1L (*Catharanthus roseus* RLK1-like) subfamily and their cognate peptide ligands, RALF (Rapid Alkalinization Factor) peptides, have been characterized in quinoa (Jiang et al., 2022). Functional investigation demonstrated that CqRALF15 participates in salt stress response regulation, indicating that RLK-mediated extracellular signal perception contributes to quinoa’s salinity response. RLK signaling may feed into MAPK cascades and Ca²^+^ signaling pathways, though the specific downstream connections in quinoa remain to be established.

#### 4.2.4 Flooding stress response pathways

Flooding stress activates specific signaling cascades in quinoa that are distinct from those induced by drought or salinity. Combined transcriptome and metabolome analyses demonstrated that flooding substantially enhances flavonoid biosynthesis in quinoa seedlings, with the accumulation of specific flavonoid compounds correlating with improved flooding tolerance (Wang et al., 2025b). Flavonoids contribute to flooding resistance through multiple mechanisms: direct ROS scavenging, modulation of ethylene signaling, and reinforcement of cell wall integrity. Transcriptomic and metabolomic analyses further identified the glycolytic, galactose, pentose phosphate, and citric acid pathways as key metabolic routes activated in quinoa’s flooding response (Guo et al., 2022), consistent with energy metabolism reprogramming under hypoxia.

## 5 Transcriptomic and multi-omics insights

**Figure 3:**
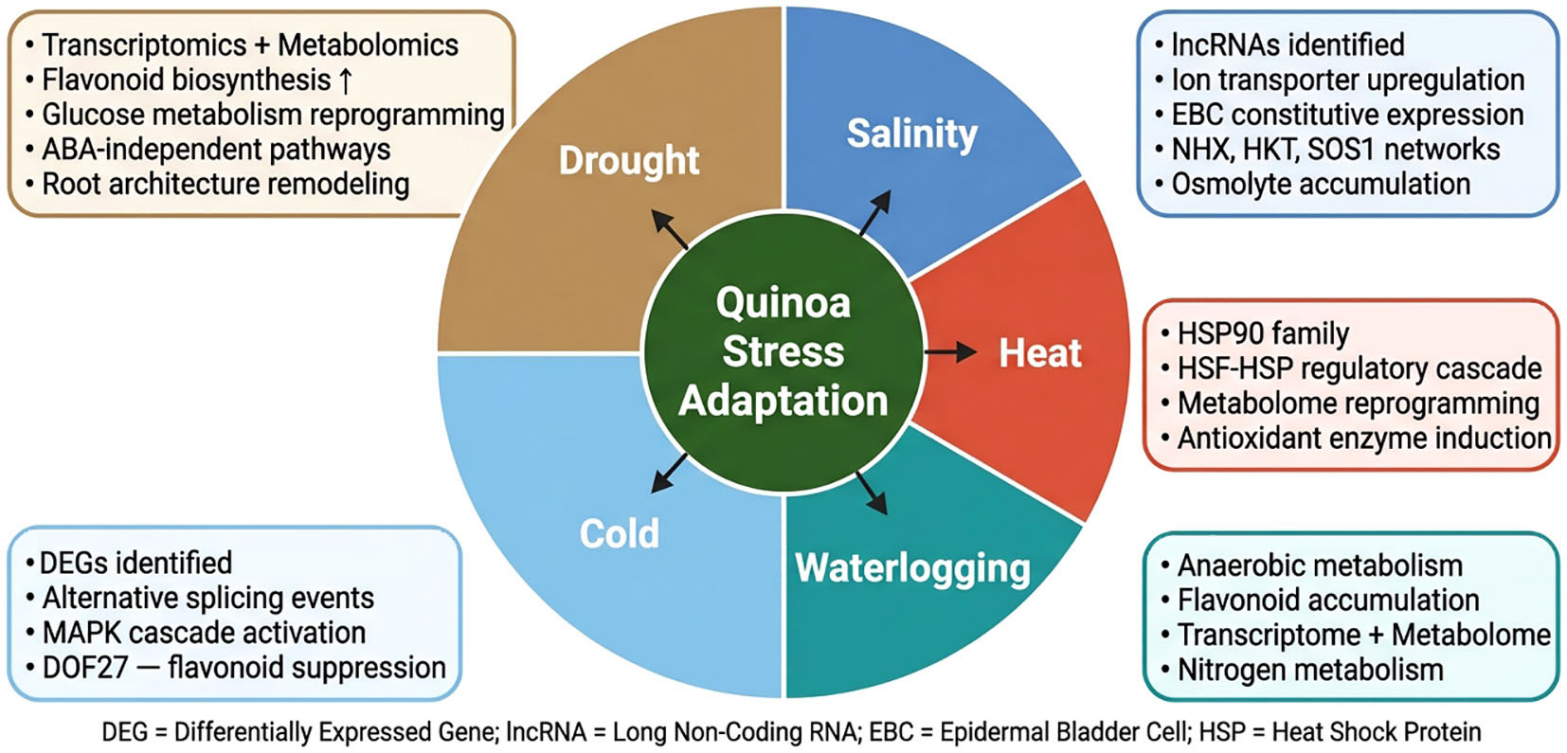
Multi-omics landscape of quinoa abiotic stress responses and cross-layer regulatory connections. This hub-and-spoke wheel diagram summarizes the key mechanistic insights emerging from multi-omics studies for each of five abiotic stresses, emphasizing the functional connections between omics layers rather than individual datasets. Drought: transcriptomic upregulation of ABA signaling components is functionally linked to metabolomic accumulation of proline and soluble sugars, demonstrating a transcriptome-to-metabolome connection that drives osmotic adjustment. Salinity: transcriptomic changes in ion transporter genes (NHX, HKT, SOS1) connect to the EBC-based ion sequestration system; lncRNA expression changes may modulate transporter gene regulation post-transcriptionally. Heat: HSF-HSP transcriptional cascades are linked to proteome protection outcomes; co-induction of USPs suggests a broader chaperone network than HSPs alone. Cold: MAPK cascade activation and alternative splicing events connect transcriptomic responses to post-transcriptional regulation; flavonoid biosynthesis upregulation links transcriptomics to metabolomic antioxidant defense. Waterlogging: flavonoid biosynthesis and ethylene signaling emerge as key integrative nodes connecting transcriptomic and metabolomic responses to flooding tolerance.

Advances in high-throughput sequencing and mass spectrometry technologies have enabled comprehensive characterization of quinoa’s stress responses at the transcriptome, metabolome, and proteome levels. The power of multi-omics approaches lies not in the parallel accumulation of data from each layer, but in their functional integration: connecting transcriptional changes to metabolic outcomes reveals the regulatory logic of stress adaptation in ways that single-omics approaches cannot (**Figure 3**). The following subsections synthesize key findings from multi-omics studies, emphasizing the mechanistic connections between omics layers rather than cataloguing individual datasets.

### 5.1 Drought stress responses

Combined transcriptomic and metabolomic studies have provided mechanistically integrated insights into quinoa’s drought tolerance. In drought-tolerant genotypes, transcriptomic and metabolomic analyses have identified starch and sucrose metabolism and flavonoid biosynthesis as key pathways linking gene expression changes to metabolite accumulation (Huan et al., 2022). This transcriptome-to-metabolome connection is particularly clear in studies comparing tolerant and sensitive genotypes: tolerant accessions show coordinated upregulation of both biosynthetic genes and the corresponding metabolite accumulation, while sensitive genotypes show a more limited integrated response (Wang et al., 2024b). Candidate drought tolerance genes identified through transcriptomics, including those involved in glucose metabolism reprogramming, have been validated by yeast expression systems, providing a functional bridge between expression data and biochemical activity (Wang et al., 2024a).

Molecular and physiological characterization of contrasting quinoa genotypes under water deficit conditions further revealed that drought-tolerant genotypes consistently maintained higher activities of SOD, CAT, and POD, along with greater accumulation of proline and soluble sugars, compared with sensitive genotypes (Zhu et al., 2024). These findings highlight the importance of integrating enzymatic activity data with transcriptomic and metabolomic profiles to obtain a complete picture of stress adaptation, and underscore the value of genotype comparisons for identifying the most functionally relevant regulatory differences.

### 5.2 Salt stress responses

Transcriptome profiling of contrasting quinoa genotypes under salt stress identified candidate genes associated with tolerance, including ion transporters, osmolyte biosynthesis enzymes, and stress-responsive transcription factors that were differentially expressed between tolerant and sensitive accessions (Shi and Gu, 2020). A transcriptome dynamics study uncovered a large number of long non-coding RNAs (lncRNAs) in quinoa, a substantial proportion of which exhibited salt-responsive expression patterns (Luo et al., 2022). These lncRNAs may modulate stress responses through post-transcriptional mechanisms — such as acting as competing endogenous RNAs or regulating chromatin accessibility — though functional validation of individual lncRNAs in quinoa is currently lacking, and their regulatory roles remain largely inferential. Connecting lncRNA expression changes to their putative target ion transporter genes (e.g., NHX, HKT, SOS1) through co-expression network analysis represents a promising avenue for future mechanistic studies.

At the protein level, combined transcriptomic and proteomic analyses of quinoa under ethylene and salt stress revealed that ethylene signaling modulates the expression of ion transporter genes and antioxidant enzymes, providing a functional link between hormone signaling and ionic homeostasis (Ma et al., 2021). Single-cell transcriptomic analysis of quinoa salt bladders has begun to reveal the developmental trajectory and cell-type-specific gene expression patterns that underlie EBC function (Liu et al., 2024), offering a new level of resolution for understanding how the EBC system is established and maintained.

### 5.3 Cold stress responses

Comparative transcriptomic analyses between cold-tolerant and cold-sensitive cultivars identified substantial numbers of differentially expressed genes in response to cold stress during seed germination, with the exact numbers varying considerably by genotype, developmental stage, and experimental conditions (Fu et al., 2025; Zheng et al., 2022). Pathway enrichment analysis consistently revealed that MAPK signaling, oxidative phosphorylation, and flavonoid biosynthesis pathways are specifically enriched in cold-tolerant genotypes, suggesting that enhanced antioxidant capacity and secondary metabolite accumulation are key determinants of cold tolerance. Full-length transcriptome sequencing further revealed extensive alternative splicing events under cold stress, with cold-tolerant cultivars showing more pronounced splicing changes than sensitive ones (Zheng et al., 2022). This finding connects the transcriptomic response to post-transcriptional regulation, suggesting that alternative splicing may generate stress-adapted protein isoforms that contribute to cold tolerance — a mechanistic layer that warrants further investigation.

A persistent challenge in quinoa multi-omics research is that most published studies report transcriptomic and metabolomic data in parallel without experimentally validating the causal connections between them. True cross-omics integration — for example, demonstrating that a specific transcription factor directly regulates a metabolic pathway through ChIP-seq validation, or that a lncRNA modulates transporter gene expression through loss-of-function experiments — remains rare in the quinoa literature. This gap reflects the early stage of functional genomics in this non-model species, where genome editing and transgenic validation tools have not become available.

## 6 Conclusions and perspectives

The molecular, physiological, and biochemical responses of quinoa to diverse abiotic stresses demonstrate that this crop possesses a multi-layered stress tolerance system. At the protein level, quinoa deploys an extensive repertoire of protective proteins — including HSPs, USPs, LEA proteins, and an expanded GPX family — that operate synergistically to preserve cellular homeostasis under adverse conditions. The EBC system represents a genuinely distinctive structural and molecular adaptation that concentrates ion transport machinery in specialized epidermal cells, providing a salt sequestration capacity that exceeds that of most other halophytes. At the transcriptional level, a diverse array of TF families orchestrates stress-responsive gene expression, with several families showing quinoa-specific expansions. However, gene family expansion alone does not constitute functional specialization. Demonstrating quinoa-specific TF functions requires direct experimental validation, which is currently available for only a small number of members. At the signaling level, interconnected hormone-mediated and non-hormone cascades integrate stress perception with adaptive responses through extensive crosstalk, with ABA, MAPK, and Ca²^+^/CPK pathways serving as central hubs.

Despite this substantial progress, important knowledge gaps remain, and we identify four priority areas for future research.

First, functional validation of candidate genes is urgently needed. The majority of stress-associated gene families identified through genome-wide analyses have not been experimentally validated in planta. Future studies should prioritize loss-of-function analysis, overexpression lines, and protein interaction studies for the most promising candidates identified by multi-omics approaches. The link between abiotic stress and secondary metabolite accumulation — particularly flavonoids, betalains, and saponins — deserves particular attention, as these compounds may serve both protective and signaling roles under stress. Identification of polyresistance genes that confer tolerance to multiple stresses simultaneously would be especially valuable for breeding applications given that field conditions typically involve concurrent stresses. Furthermore, the synergistic effects of regulatory pathway interactions — specifically the convergence of ABA, MAPK, and Ca²^+^/CPK signaling on shared downstream targets — represent key integrative nodes whose combinatorial logic has not yet been systematically investigated in quinoa and warrants dedicated mechanistic study.

Second, post-translational and epigenetic regulatory mechanisms remain largely unexplored in quinoa. At the post-translational level, phosphorylation of TFs and signaling components by SnRK2 and MAPK kinases, ubiquitin-mediated proteasomal degradation of stress regulators, sumoylation, and glycosylation are likely to play important roles in fine-tuning stress responses, but have not been systematically studied in quinoa. Beyond these classical PTMs, novel acylation modifications — including lactylation, crotonylation, and β-hydroxybutyrylation — have recently been identified as regulators of stress responses in other organisms and may operate in plants as well. At the epigenetic level, DNA methylation changes, histone modifications (H3K4me3, H3K27me3), and RNA modifications (m^6^A) may contribute to stress memory and transgenerational adaptation in quinoa. Stress memory — the capacity of plants to mount a faster and stronger response upon repeated stress exposure — is an epigenetically regulated phenomenon that has been documented in Arabidopsis and crop species but remains entirely uncharacterized in quinoa, representing a particularly promising and largely unexplored dimension of its stress response.

Third, advanced genetic transformation and gene editing technologies will be essential for functional genomics and crop improvement in quinoa. Current Agrobacterium-mediated transformation protocols remain genotype-dependent and relatively inefficient in quinoa. The development of genotype-independent transformation methods — including those based on developmental regulators (e.g., Baby Boom, Wuschel) would greatly accelerate functional validation studies across diverse quinoa accessions. *Rhizobium rhizogenes*-mediated hairy root transformation offers a rapid and genotype-flexible alternative for gene function studies, particularly for root-expressed stress-tolerance genes, and its application in quinoa deserves further development. The expanding toolkit of genome editing technologies— CRISPR-Cas for loss-of-function analysis, its derived base editing for single-nucleotide changes, and prime editing for targeted sequence modifications — provides complementary instruments for both mechanistic studies and targeted improvement of stress tolerance traits. Their application in quinoa is expected to expand rapidly as transformation efficiency improves.

Fourth, next-generation omics technologies will provide new levels of resolution for understanding quinoa stress responses. At the genome level, long-read sequencing technologies will improve the characterization of alternative splicing events and lncRNA structures that contribute to stress responses. At the spatial and single-cell level, spatial transcriptomics will enable cell-type-specific gene expression profiling in EBCs and other specialized tissues without the need for cell isolation, while single-cell RNA sequencing will reveal the cellular heterogeneity of stress responses and identify rare cell populations with specialized functions. At the epigenetic modification level, chromatin accessibility profiling (ATAC-seq) and ChIP-seq for stress-responsive histone marks will connect epigenetic changes to transcriptional outputs, enabling systematic mapping of the regulatory landscape under stress. At the protein level, proteomics approaches capable of mapping PTMs at the proteome scale — including phosphoproteomics and acylome profiling — will bridge the gap between transcriptional changes and protein-level regulation, revealing the post-translational logic of stress adaptation. Integration of these multi-omics layers within a systems biology framework will ultimately be required to fully understand how quinoa coordinates its exceptional stress resilience — and to translate that understanding into climate-adaptive crop improvement strategies.
